# Extinction Risk Escalates in the Tropics

**DOI:** 10.1371/journal.pone.0003886

**Published:** 2008-12-10

**Authors:** Jana C. Vamosi, Steven M. Vamosi

**Affiliations:** Department of Biological Sciences, University of Calgary, Calgary, Canada; University of Sheffield, United Kingdom

## Abstract

The latitudinal biodiversity gradient remains one of the most widely recognized yet puzzling patterns in nature [1]. Presently, the high level of extinction of tropical species, referred to as the “tropical biodiversity crisis”, has the potential to erode this pattern. While the connection between species richness, extinction, and speciation has long intrigued biologists [2], [3], these interactions have experienced increased poignancy due to their relevancy to where we should concentrate our conservation efforts. Natural extinction is a phenomenon thought to have its own latitudinal gradient, with lower extinction rates in the tropics being reported in beetles, birds, mammals, and bivalves [4]–[7]. Processes that have buffered ecosystems from high extinction rates in the past may also buffer ecosystems against disturbance of anthropogenic origin. While potential parallels between historical and present-day extinction patterns have been acknowledged, they remain only superficially explored and plant extinction patterns have been particularly neglected. Studies on the disappearances of animal species have reached conflicting conclusions, with the rate of extinction appearing either higher [8] or lower [9] in species richness hotspots. Our global study of extinction risk in vascular plants finds disproportionately higher extinction risk in tropical countries, even when indicators of human pressure (GDP, population density, forest cover change) are taken into account. Our results are at odds with the notion that the tropics represent a museum of plant biodiversity (places of historically lowered extinction) and we discuss mechanisms that may reconcile this apparent contradiction.

## Introduction

The tropical biodiversity crisis has been escalating for decades. We know that an ever-increasing percentage of threatened species of birds, mammals and conifers are found in the Neotropics [10]. While it is appreciated that this extinction has both natural and anthropogenic causes, disentangling the contribution of each to the demise of any particular species has proven immensely challenging [11]–[13]. Whether tropical species are more innately vulnerable to extinction can only be determined if we concurrently assess the confounding influences of human impact, which may also exhibit a latitudinal gradient. Tropical species may instead be more resilient to extinction, a factor that may have played an important role in the formation of the latitudinal biodiversity gradient [6], [14]. If human disturbance can be assumed roughly equivalent to natural catastrophes that have occurred over evolutionary time scales, knowledge of the distribution of susceptibility to extinction in the present may reveal important features of extinction rates that relate to latitudinal diversity.

Examined over large times scales, extinction rates have been described as higher and lower in tropical biomes. On the one hand, tropical climates have been considered to be relatively old, benign, and stable (with reduced climatic oscillations) compared to temperate ones, making reduced extinction rates a possible factor producing the latitudinal diversity gradients in plant and animal clades–the “museum” hypothesis [4], [5], [15]–[18]. Once established, the additional species richness of the tropics may provide some buffering from further disturbance, a potential ecosystem function conveyed by biodiversity that further reduces extinction rates [19]. If tropical environments are indeed buffered somehow from extinction, then we should observe that, for any given amount of human impact, the tropics should experience a lowered per-species extinction rate (i.e., an important interaction effect between human impact and ecology [11]). While at odds with the processes that would produce a latitudinal biodiversity gradient, some studies indicate a positive association between speciation rates and extinction rates [20], indicating that tropical species may actually be more susceptible to disturbance. Geographical range studies show tropical species have smaller ranges and population densities [15], [21]. The greater endemicity and lower population size of tropical species could make them more susceptible to the types of fragmentary disturbance inflicted by deforestation and urban development. Whether present day extinction is occurring in areas that may have experienced low levels of natural extinction historically (i.e., the tropics) would have ramifications on the predicted loss of evolutionary history [5], [22] and proper adjudication of conservation resources [23].

The high numbers of species in tropical countries will inevitably increase the likelihood that they harbor high numbers of threatened species. The reported lack of congruence between hotspots in the absolute numbers of threatened species and hotspots in total species richness indicates that intrinsic susceptibilities of species to extinction or extrinsic risk factors such as deforestation are not distributed uniformly upon the globe [8], [11]. Previous studies have then confounded any intrinsic extinction risk that is correlated with latitude with extrinsic risk by not controlling for relationships with latitude, or between metrics of extrinsic risk. Thus, to explore intrinsic differences in the species that inhabit tropical versus temperate countries we must concurrently investigate human impact factors on the proportion of threatened species relative to the underlying species richness of the area. Getting estimates of this kind for plants have been historically hindered by the paucity of data from tropical countries on the estimated number of plant species in their flora and, even more importantly, on the estimated number of threatened plant species in their flora [24]. However, increasingly complete datasets of this kind are now available to make the comparisons needed in order to extract the process of extinction from the pattern. Here, we employ a log-odds approach [25] to examine whether: (1) countries with high background species richness are unduly challenged to maintain plant species richness because of inherently high extinction rates or (2) local species diversity acts to buffer further extinction producing a pattern whereby tropical countries are predicted to have low extinction risk relative to the number of species present (while still having albeit higher absolute numbers of threatened species), while taking into account the effects of deforestation rates, population density, and per-capita gross domestic product (GDP). The determination of the relative importance of extrinsic (human-induced) versus intrinsic (species traits/diversity) factors on the distribution of present-day plant extinction hotspots will help direct our limited resources to where they are most needed.

## Results

**Figure 1:**
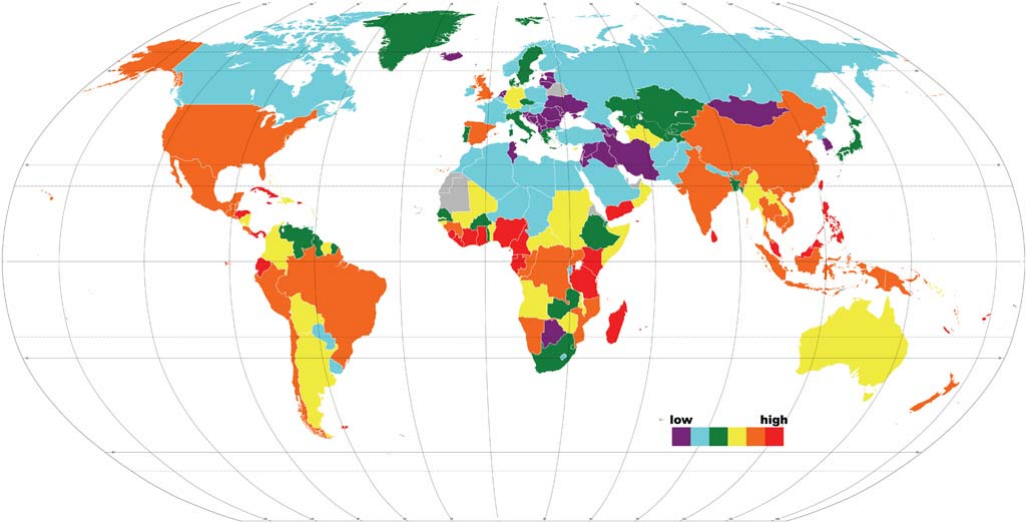
Map of the threat in the countries of the world. Most mainland countries range from every vascular plant species having a one in ten chance (red) to a one in 10000 (purple) chance of being at-risk of extinction.

**Figure 2:**
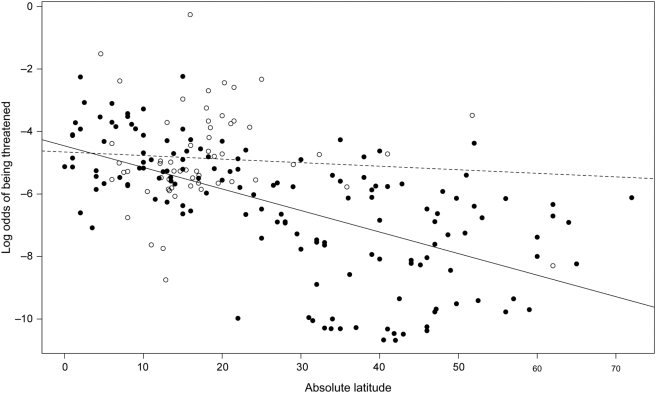
Relationship between latitude and log-odds of being threatened for mainland (filled circles, solid line) and island (open circles, dashed line) countries.

The odds of finding threatened plant species vary widely from country to country (Fig. 1), and sometimes even among neighboring countries. However, correlograms revealed positive correlations (coefficients: 0.4–0.95) overall for pairs of countries close to one another (i.e., within 25° of one another; see also Fig. 1), with this pattern being robust to the number of bins considered (S. Vamosi, unpubl. data). Accounting for spatial autocorrelation (see Methods), there was a significant interaction between the effects of absolute latitude and country type (mainland versus island) on log-odds threatened (*t*_1,205_ = –2.43, *P* = 0.016), whereas neither main effect was significant on its own (absolute latitude: *t*_1,205_ = –0.015, *P* = 0.45; country type: *t*_1,205_ = 0.07, *P* = 0.89). Visual inspection of the resulting plot reveals that log-odds threatened declined with latitude in mainland countries, whereas there was no relationship between these variables in island countries (Fig. 2). Log-odds threatened was similar for low latitude mainland and island countries, whereas high latitude countries tended to have lower log-odds than comparable island countries. Another point that emerges from this plot is that, because most island nations are found at low latitudes (i.e., <25° north or south of the equator), island countries tend to have rather high proportions of their floras threatened.

**Figure 3:**
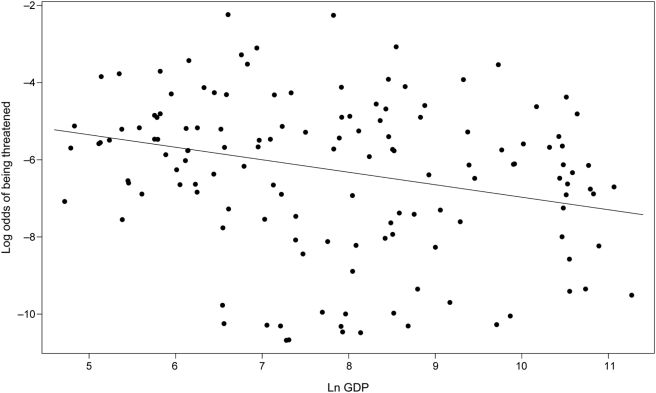
Relationship between GDP and log-odds of being threatened for mainland countries.

Restricting our attention to mainland countries, and continuing to account for spatial autocorrelation, we find that two of our variables are significantly associated with log-odds threatened: absolute latitude (*t*_1,135_ = −8.17, *P*<0.0001) and ln-transformed per-capita GDP (*t*_1,135_ = 2.38, *P* = 0.019). High latitude mainland countries had lower log-odds threatened than low latitude countries (see Fig. 2). Additionally, mainland countries with high GDP had lower log-odds threatened than countries with low GDP (Fig. 3). In other words, although there are associations between latitude and (1) deforestation rates (*r* = 0.39; *P*<0.0001) and (2) species richness (*r* = −0.49, *P*<0.0001), but not (3) population density (*r* = 0.03, *P* = 0.72), none of these variables significantly impact the proportion of threatened species in a flora. Thus, the pattern that a tropical country will have a higher proportion of threatened species than will one closer to the poles is not simply mediated through the anthropogenic factors explored here, with the exception of GDP. Surprisingly, species diversity appeared to have no significant buffering effect against disturbance, indicating that the strong association between species richness and latitude is not responsible for the observed pattern. Although per-capita GDP was retained in our minimal adequate model, two points are worth mentioning. First, the significance level for GDP was markedly lower than for latitude. Second, residual regression revealed that countries with above average GDP values (i.e., greater than predicted for their latitude) were not associated with lower log-odds threatened (GLS; *t*_1,136_ = 1.95, *P* = 0.29). In short, our results indicate that, because latitude is the strongest determinant of log-odds threatened, species in tropical countries appear have a greater natural susceptibility to extinction.

## Discussion

Our findings indicate that species vary in their natural susceptibility to extinction via disturbance, with plant species inhabiting tropical countries being more sensitive to a given degree of human impact. These results are in agreement with previous studies in non-angiosperm species [10]: threat is concentrated in species-rich nations and the increased level of threat is above that expected due to the increased number of species located in these nations. We find different patterns between island and mainland nations with the relationship between latitude and threat being much stronger for mainland countries; island nations on the other hand exhibit high levels of threat regardless of latitude. Because geographical range strongly determines the extinction risk of any particular species [25] and levels of endemism determines the proportion of at-risk species in any one country [18], isolated island nations with high levels of endemic species are predisposed to high extinction rates.

Contrary to previous reports [10] we find that the risk of extinction is higher in biodiversity hotspots regardless of, and not because of, the influences of human impact. Even when we include the effects of differential amounts of human disturbance in the model, most human impact measures were not significant determinants of the proportion of at-risk plant species per country. While human impact was observed to have a surprisingly small effect, there were still sharp delineations in the proportion of species at risk across national boundaries (Fig. 1). However, it is important to not immediately reduce these to differences in socioeconomic factors, as even the metric most closely aligned with wealth (per-capita GDP) had a minimal effect (Fig. 3) when compared to the effects of latitude. In other words, while humans cannot be excused from causing the pattern of increased extinction in the tropics, it is not an increased degree of human impact in tropical regions *per se* that produces the pattern. Rather, species in tropical regions appear more susceptible to a given amount of disturbance. The departure from previous findings may be due to the fact that the factors influencing extinction rates in plants differ from that in bird and amphibian species. Also, our results are influenced by the fact that we examined *per-species risk* as opposed to the absolute number of threatened species per country, thus taking into account the likelihood that the number of threatened species will be influenced by the underlying latitudinal gradient in species richness (i.e., species-rich nations are prone to harboring high numbers of threatened species simply because they have more species in general).

In agreement with previous reports [10] we posit that present day imminent plant extinctions may appear localized within different areas compared to the “sensitive species” lost during historical extinctions. Thus, extinction rates appear to be highest where extinction rates are thought to have been lowest in the past. This seems to refute the “museum” theory that the stable, benign climate of the tropics results in reduced extinction [2], 5. However, our findings would be in agreement with other paleontological [20] and phylogenetic [4] studies that have found that speciation rates and extinction rates are positively correlated. We posit that the same process that drives speciation may also drive extinction. This would occur if increased mutagenesis, lineage splitting, and subsequent gene fixation [3], [26] typically produced nascent species with smaller ranges, lower abundance and lower genetic diversity that are inherently susceptible to disturbance and Allee effects [27]. Should these processes be more common in the tropics, the latitudinal biodiversity gradient may be partly a result of tropical species experiencing fewer mass extinction events yet higher background rates of extinction. Thus, while tropical climates are stable over long periods of time (lowered climatic cycles), the high speciation rate in the tropics generates many species with low range sizes and low population sizes (i.e., many species susceptible to habitat perturbations).

Whether the disturbances produced by human impact can possibly reflect what tropical areas experienced in terms of disturbance over evolutionary time scales is debatable. Should human disturbance not be providing a window into natural background extinction dynamics within the tropics, we need to carefully examine what the disproportionate loss of tropical species will mean to conservation of phylogenetic diversity. If tropical ecosystems harbor many “museum” species, concentration of extinction within the tropics could result in the loss of many species with high evolutionary history [28]. Furthermore, there is a risk that extinction in the tropics will start extinction cascades [29]. It has been hypothesized that increased species diversity may drive the evolution of specialization, producing communities with many species precariously dependent on interspecific interactions [22]. However, we found that species diversity was not a significant predictor of the level of the extinction risk, a finding not consistent with the idea that local species richness influences whether any one species will become threatened. Should tropical climates harbor ancient clades, the additional evolutionary history within these areas warrants additional conservation concern [30] and human-induced extinction in these areas will be even more grave than previously thought.

## Materials and Methods

Unlike with the grid data of birds and mammals where the locations of threatened species are estimated to 1° latitude resolution, we have used data for countries, the only resolution possible for upwards of 350,000 angiosperm species. Information was gathered from the 2007 IUCN Red List on the number of threatened species in 181 countries of the world. Because only a portion of the taxa represented in the 1997 Red List have had their status re-classified according to new guidelines of the 2006 Red List, the threat status of species listed in the 1997 and 2006 versions were combined [31]. When a country was listed in both versions, we recorded the number of threatened species that was higher, yet likely these values still represent underestimates in many cases. Several countries have no estimates of the number of threatened species (e.g. Botswana) due to inadequate information on the flora. The IUCN threat categories (v3.1) used include Vulnerable (VU), Endangered (EN), Critically Endangered (CR) and Extinct (EX), while Least Concern (LC) and Near Threatened (NT) were combined with “not threatened” (nt). Species richness of the flora within the countries was obtained from the IUCN list, and missing information was searched and gathered from published sources where required. Data for absolute latitudinal midpoint, per-capita GDP, population density, and percent forest cover change of each nation were acquired from published online sources (e.g., CIA World Factbook (www.cia.gov/library/publications/the-world-factbook/) for per-capita GDP, population density and latitude, Global Forest Watch (www.globalforestwatch.org) for percent forest cover change and BioMaps (www.biologie.uni-hamburg.de/b-online/bonn/Biodiv_mapping/biomaps.htm) for mean alpha-diversity (background species richness).

Species were coded as either threatened (VU, EN, CR) or not threatened (LC, NT, nt) and logistic regression was then used to regress the binary codes against the log-species richness (SR) of the countries [32]. Logistic regression is essentially ordinary regression using the logit, or the log-odds of any particular species within a family being threatened (*T*), as the response variable. Thus, logit(*T*) = log(prob(*T*)/(1−prob(*T*)) = *a*+*b* ln(SR). Logit (or log-odds) values of individual countries are displayed in Fig. 1 and these logit values are plotted against ln-SR in Fig. 2. As in other studies [25], logit transformation converted a difficult variable into one that was reasonably tractable.

### Statistical Analyses

To investigate possible mechanisms underlying the global patterns in log-odds threatened (Fig. 1), we conducted a series of analyses. First, we addressed whether log-odds threatened exhibited significant spatial autocorrelation using the library ‘spatial’ in R 2.7.0 [33], following the methods described by Crawley [34]. Correlograms revealed positive correlations (coefficients: 0.4–0.95) for pairs of countries close to one another (i.e., within 25° of one another; see also Fig. 1), with this pattern being robust to the number of bins considered (S. Vamosi, unpubl. data). Therefore, to account for spatial autocorrelation, we applied a generalized least squares (GLS) approach, which allows the user to input the spatial coordinates of replicates (countries) and to specify the within-group correlation structure, using the ‘nlme’ library in R. A limitation to this approach is that it cannot handle missing values; therefore, all subsequent analyses presented include only those countries for which all we had data entries for all included variables. Preliminary data analyses revealed that the GLS models produced similar effect sizes, but more conservative significance values, than comparable generalized linear models that did not account for spatial autocorrelation (S. Vamosi, unpubl. obs.).

Second, we explored potential differences between mainland and island countries. All else being equal, extinction rates are expected to be higher on islands [35]. We analyzed the main effects of absolute latitude and country type (*n*_M_ = 149; *n*_I_ = 60), and the interaction between the two factors, on log-odds threatened.

Third, we examined the effects of absolute latitude, background diversity, GDP, % forest change, and population density on log-odds threatened. We present only the results of analyses with mainland countries, because the sample size for island countries for which we had data for all five variables was low (*n* = 42). Although some of the variables were correlated with one another, we applied a (spatially explicit) multiple regression approach, rather than a residual regression one, as recommended by Freckleton [36]. Because of the number of higher-order interactions with five main effects, we applied a modified version [37] of the typical model simplification approach, in which one starts with the full model and removes least significant highest-order terms (e.g., the five-way interaction) singly in a stepwise manner [38]. Here, we calculated the AIC scores for five different models, each progressively reduced in terms of number of parameters. The first model was the full model, which included all main effects and all higher-order interactions (i.e., 10 two-interactions, 10 three-way interactions, five four-way interactions, and one five-way interaction). The remaining models were subsets of the full model, with each removing all interactions in the highest remaining category. For example, the second model included the main effects and all higher-order interactions but the five-way interaction. Model simplification produced lower AIC scores in all cases. We then resumed the standard model simplification approach, removing least significant main effects from the fifth model (i.e., the one that contained only the five main effects) until we were left with the minimum adequate model (i.e., one with only significant terms). To confirm that our final model was robust to this approach, we then compared its AIC with that of a model that included an interaction between the two remaining main effects. This model had a higher AIC (546.12) than the minimal adequate model (542.77); therefore we present the results of the model with only the two main effects. Additionally, the inferences from this model are in close agreement with the outcome of a reduced model in which we included latitude, background diversity and PC1 of a principal components analysis that combined the “human pressure variables” (i.e., GDP, population density, % forest change) into one variable.
